# Radon Exposure Assessment: IoT-Embedded Sensors

**DOI:** 10.3390/s25196164

**Published:** 2025-10-05

**Authors:** Phoka C. Rathebe, Mota Kholopo

**Affiliations:** Department of Environmental Health, Faculty of Health Sciences, Doornfontein Campus, University of Johannesburg, Johannesburg 2006, South Africa; motaliok@gmail.com

**Keywords:** radon exposure, Internet of Things (IoT), environmental monitoring, lung cancer, calibration, real-time data, risk mitigation, Wireless Sensor Networks, AI-driven forecasting

## Abstract

Radon exposure is the second leading cause of lung cancer worldwide, yet monitoring strategies remain limited, expensive, and unevenly applied. Recent advances in the Internet of Things (IoT) offer the potential to change radon surveillance through low-cost, real-time, distributed sensing networks. This review consolidates emerging research on IoT-based radon monitoring, drawing from both primary radon studies and analogous applications in environmental IoT. A search across six major databases and relevant grey literature yielded only five radon-specific IoT studies, underscoring how new this research field is rather than reflecting a shortcoming of the review. To enhance the analysis, we delve into sensor physics, embedded system design, wireless protocols, and calibration techniques, incorporating lessons from established IoT sectors like indoor air quality, industrial safety, and volcanic gas monitoring. This interdisciplinary approach reveals that many technical and logistical challenges, such as calibration drift, power autonomy, connectivity, and scalability, have been addressed in related fields and can be adapted for radon monitoring. By uniting pioneering efforts within the broader context of IoT-enabled environmental sensing, this review provides a reference point and a future roadmap. It outlines key research priorities, including large-scale validation, standardized calibration methods, AI-driven analytics integration, and equitable deployment strategies. Although radon-focused IoT research is still at an early stage, current progress suggests it could make continuous exposure assessment more reliable, affordable, and widely accessible with clear public health benefits.

## 1. Introduction

Radon is a leading cause of lung cancer after tobacco smoking and continues to pose a major, yet preventable, global health burden. However, progress in reducing the risk is limited by the availability of mitigation technologies rather than by the ongoing challenges in radon monitoring. Radon is classified as a Group 1 carcinogen by the International Agency for Research on Cancer (IARC) and migrates into indoor environments primarily via soil gas intrusion, fissures in building foundations, and radon-emitting construction materials [1,2,3,4]. Its burden is well documented across various regions. The U.S. Environmental Protection Agency (EPA) attributes approximately 21,000 lung cancer deaths annually to radon [4], while European meta-analyses show that every 100 Bq/m^3^ increase in radon concentration is associated with an approximately 8.4% increase in lung cancer risks [5]. In the Middle East, exposure in several provinces exceeded the World Health Organization (WHO) and EPA standards, with household doses of up to 5 mSv per year [6]. These data emphasize radon’s role as a pervasive, independent carcinogen. Although scientific evidence links radon exposure to adverse respiratory health outcomes, monitoring efforts remain fragmented and inconsistent across regions. Passive detectors, such as alpha track and charcoal canisters, are cost-effective and widely accessible; however, they provide only retrospective measurements and are incapable of capturing temporal variations or accounting for environmental factors influencing radon exposure risk [7,8]. Continuous radon monitors (CRMs) offer better temporal resolution, but are expensive, complex to set up, and not suitable for widespread use in homes, schools, or workplaces [9]. Additionally, current calibration and validation standards were designed for passive or manually operated devices, and do not yet account for telemetry, automated sampling, or embedded processing, which are key features of modern monitoring tools [10,11]. This monitoring gap presents a significant preventability challenge. Although mitigation techniques such as sub-slab depressurization and enhanced ventilation have demonstrated efficacy [12], the absence of affordable and scalable monitoring systems results in undetected and consequently unmitigated radon exposures.

The rapid growth of the Internet of Things (IoT) offers a timely chance to address this gap. IoT-based radon monitoring systems use embedded micro-controllers, affordable sensors, and long-range wireless connectivity (e.g., LoRaWAN, NB-IoT) combined with edge-to-cloud analytics to deliver continuous, distributed, and high-resolution exposure data [13,14,15,16]. Pilot deployments have demonstrated potential. For example, IoT networks in Tehran schools identified persistent basement hotspots [17], prototypes in Portugal enabled real-time mapping through WebGIS dashboards [13], and experimental devices in Canada and the United States revealed temporal links between ventilation cycles and radon peaks [18,19]. Still, there are several challenges that need to be addressed. Low-cost sensors, like metal oxide semiconductors, can be impacted by human drift [17]. Calibration protocols also differ significantly [13,18], and the issue of power autonomy in areas with limited infrastructure or in rural settings still needs to be resolved [15,20]. Furthermore, concerns about data ownership, privacy, and regulatory acceptance add to the challenges [21]. These challenges show both the potential and vulnerability of IoT radon systems, making a thorough, integrative review crucial at this point. This review is driven by the urgent need to address the limitations of legacy radon monitoring while taking advantage of the advances in IoT-enabled sensing. It aims to (i) synthesize the evidence base on IoT radon systems, (ii) show how IoT can overcome the main shortcomings of traditional monitoring, such as temporal blindness, sparse coverage, delayed feedback, and unequal access, and (iii) identify the validation, standardization, and governance needs required to scale prototypes into a reliable public health infrastructure [10,11,13,15,17,18,19,22,23,24]. By placing radon monitoring within the broader context of environmental IoT applications, this review highlights that effective radon control is not only a technical challenge but a matter of health equity and policy readiness.

The review is organized as follows. Section 2 outlines the methodology and evidence synthesis undertaken to compile this manuscript. Section 3 presents a comparative analysis of the five IoT-radon studies identified, highlighting their technical designs, calibration practices, and field performance. Section 4 provides the principles and components of IoT sensing technologies, including detection methods, embedded architecture, and communication protocols. Section 5 discusses deployment strategies from single buildings to community networks and draws lessons from related environmental IoT domains. Section 6 examines the data lifecycle, from edge processing to cloud analytics, visualization, and exposure modeling. Section 7 addresses key challenges including validation, power and connectivity constraints, privacy, and equity. Section 8 looks ahead to future directions, including next-generation sensors, predictive analytics, mitigation integration, and evolving policy frameworks. Section 9 concludes by outlining the potential for IoT-enabled monitoring to transform radon surveillance from static, fragmented testing into a proactive, real-time public health tool.

## 2. Methodology

This review followed systematic evidence synthesis methods to identify and critically assess studies on radon detection and IoT-enabled monitoring systems. The aim was to identify, assess, and combine studies related to radon detection and IoT-enabled monitoring systems. Searches were conducted in six major academic databases: Scopus, Web of Science, IEEE Xplore, PubMed, ACM Digital Library, and Google Scholar using keyword combinations such as ‘radon,’ ‘IoT,’ ‘Internet of Things,’ ‘wireless sensor network,’ “embedded systems,” and ‘real-time monitoring.” Relevant gray literature was also reviewed, including reports and standards from international organizations such as the WHO, U.S. EPA, and the International Organization for Standards (ISO/IEC) was also reviewed to minimize publication bias. The searches covered publications up to 2025. Eligible studies explicitly integrated radon sensing with IoT features such as wireless communication, cloud or edge computing, or real-time telemetry and provided sufficient methodological or deployment details for critical appraisal. Excluded works were those describing radon sensing without IoT connectivity, conceptual frameworks lacking implementation or validation, or IoT air quality studies that did not include radon.

**Figure 1:**
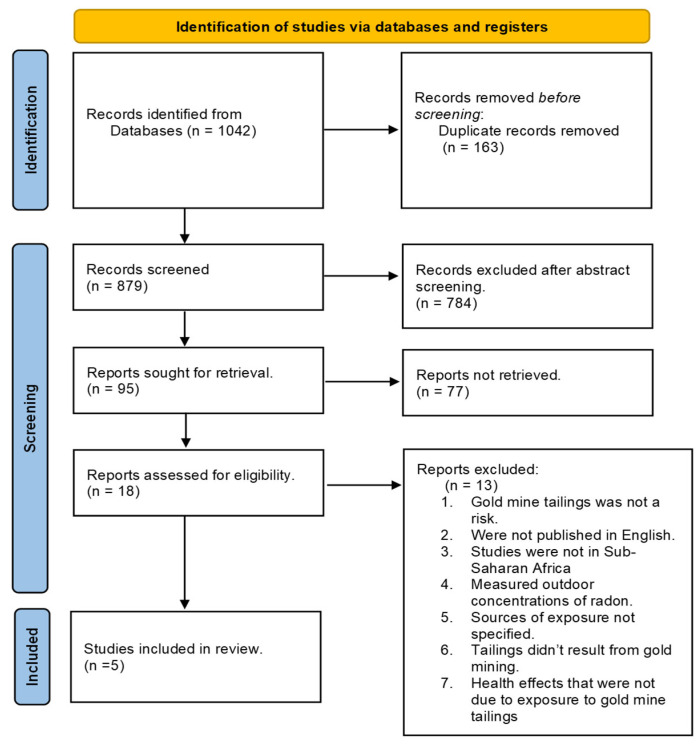
PRISMA flow diagram of the study selection. Of over 500 initial records, most were excluded because they did not incorporate IoT radon monitoring. Only five studies met the inclusion criteria, reflecting the field’s early stage.

The initial search identified over 500 records. After removing duplicates and applying the inclusion and exclusion criteria, only five primary studies remained for detailed review. Screening and selection were conducted in accordance with the PRISMA (Preferred Reporting Items for Systematic Reviews and Meta-Analyses) guidelines to ensure transparency and reproducibility. The PRISMA flow diagram (Figure 1) provides a visual summary of the process, from initial identification through screening, eligibility assessment, and final inclusion. The limited number of studies included reflects the early stage of IoT-radon research rather than the shortcoming of the search process. For each included study, data was extracted on sensor type, embedded system design, communication protocols, calibration methods, deployment context, and validation approaches. In parallel, related IoT systems (e.g., for air pollution, industrial safety, hazard monitoring) were reviewed to provide comparative insights and identify transferable solutions. This dual approach allowed for the review to synthesize both the limited radon-specific evidence and relevant technical innovations from other IoT fields.

## 3. Comparative Analysis of IoT-Integrated Radon Monitoring Studies: Core and Emerging Systems

Five primary studies met the inclusion criteria, each demonstrating how IoT technologies are being applied to radon monitoring challenges. Table 1 summarizes their technical characteristics, sensor types, calibration protocols, communication methods, and concentration ranges, highlighting trade-offs in accuracy, scalability, and field reliability. These studies provide valuable insights into the current and proposed uses of IoT systems for real-time radon monitoring. This section compares their main characteristics, with Table 1 consolidating details on sensor type, deployment setting, communication protocol, calibration and validation methods, power configuration, key results, and reported limitations. Each study contributes a distinctive perspective to the evidence base. For instance, Yousefian et al. [17] implemented a network of low-cost metal oxide semiconductor (MOS) radon sensors in 120 public schools in Tehran. Their system used NB-IoT connectivity and was calibrated with RAD7 reference monitors. The study found that basement classrooms consistently exceeded WHO safety thresholds (>100 Bq/m^3^), with sensor readings deviating by not more than ±12% from RAD7 measurements under stable conditions. However, they observed up to ±20% variability in high-humidity environments (>70%), highlighting a common calibration challenge with MOS sensors. In contrast, Dicu et al. [19], used active detectors, likely based on PIN photodiodes, in a WiFi-enabled setup across 50 homes in Alberta, Canada. The system showed a strong correlation (R^2^ = 0.87) with HVAC telemetry and seasonal radon trends, confirming its reliability in detecting winter radon spikes caused by poor ventilation. The lack of standardized calibration limited comparisons between different studies.

Lopes et al. [13] developed a system that integrates commercial sensors with a WebGIS dashboard to visualize radon levels in public buildings across Portugal in real time. Their validation involved comparing trend data with reference instruments, though it did not include comprehensive statistical calibration. The system successfully communicates risk visually, but its reliance on grid power and fixed infrastructure restricts its scalability for broader or remote use. Barros et al. [18] carried out a classroom monitoring project in Boston using open-source, consumer-grade radon sensors equipped with Wi-Fi and Bluetooth. Calibration was performed through software adjustments and user feedback, within a citizen science framework. The project was successful in promoting student engagement and enhanced ventilation practices by providing real-time feedback. Nonetheless, the reliance on low-cost sensors led to calibration drift, while differences in how users deployed the sensors resulted in inconsistent data quality. Finally, Pereira et al. [15] introduced an innovative prototype system featuring a custom LoRaWAN-enabled sensor known as RnProbe. This device incorporated edge computing capabilities for anomaly detection and was validated in laboratory settings against AlphaGuard reference instruments under controlled radon exposure. Powered by solar-assisted batteries, it showed promising potential for autonomous, long-range radon monitoring in resource-limited environments. The system remained at the prototype stage and had not yet been deployed at scale in real-world conditions.

Several key patterns emerge across the five studies. Sensor choice reflected trade-offs between cost, stability, and ease of integration. MOS sensors were often selected for affordability and rapid response, though they were sensitive to environmental conditions. In contrast, PIN-based detectors offered greater, better stability but required longer integration times, and were less sensitive at lower concentrations. Deployment environments vary widely, from educational institutions to private homes and simulated settings. Connectivity reflected practical constraints: Wi-Fi was common indoors with infrastructure, while NB-IoT and LoRaWAN suited low-power or wide-area deployments. Calibration and validation were inconsistent, with few following formal standards, limiting comparability. Power setups strongly influenced system autonomy and only some studies tested energy harvesting methods like solar power. Despite these challenges, each study reported important results, from identifying hotspots to influencing ventilation strategies and public health initiatives. Together, these systems demonstrate the potential of IoT for radon exposure monitoring. Table 1 provides uniform comparison, helping to identify areas for improving design and scalability.

### Recent Advances in IoT-Integrated Radon Monitoring (2023–2025)

Along with the five core studies previously discussed, recent developments from 2020 to 2025 have significantly advanced the field of IoT-integrated radon monitoring. These newer studies show the growing maturity of IoT-radon systems and their increasing importance not only for public health but also for environmental and geophysical uses. A significant advancement in this field is the introduction of a telemonitoring system in Manado, Indonesia. This system was created to measure radon emissions along an active seismic fault line. It uses soil-based radon sensors with high resolution and advanced signal processing techniques to filter interference from thoron and actinium. Researchers analyzed the data using statistical anomaly detection models. This approach led to a prediction sensitivity of around 84% and an average early warning lead time of 2.65 days for seismic events with a magnitude of 4.5 or higher [25]. Although this study focused on a different area, it highlights the potential benefits of IoT-enabled radon monitoring for environmental hazard forecasting. A similar development is seen in the Autodigit-RAD project in Switzerland, which demonstrates the use of radon sensors integrated into a smart building system [26]. This project shows the possibility of automated, real-time radon monitoring with cloud connectivity, allowing continuous data collection and remote access for researchers and occupants. The system includes self-calibrating routines and long-term intelligence. Although tested in a single residential building, its design hints at the potential to scale across a wider range of indoor environments.

Researchers in Republic of Korea created an IoT-integrated radon measurement system designed for groundwater observatories in seismically active regions. They used narrowband IoT (NB-IoT) for low-bandwidth, long-range, and power-efficient data transmission [27]. This enables remote radon measurement collection and transmission from geographically isolated locations with minimal maintenance. Although the system was not designed for indoor use, its strong field performance under environmental stress provides valuable insights for low-resource or rural public health deployments. Li et al. [28] further expanded the physical reach of radon monitoring by introducing a new in situ underwater radon detection system that can operate autonomously for up to two weeks. This system, which functions without human intervention and maintains stable operation in submerged conditions, offers a promising model for marine and subsurface groundwater studies. Although primarily designed for environmental and geological research, the design innovations, particularly in energy autonomy and long-term data acquisition, are relevant for broader applications, including long-term indoor deployments in inaccessible or hazardous locations. For a structured comparison of these newer studies, the following table (Table 2) has been added. It complements Table 1 by summarizing key parameters, including sensor types, deployment contexts, communication protocols, validation approaches, power systems, and observed limitations.

Together, these studies show a clear progression in IoT systems from static, indoor-focused platforms to more dynamic, autonomous, and environmentally resilient architectures. They also highlight a shift in research priorities from simple detection to predictive analytics, long-range telemetry, and low-maintenance operation. Notably, they show how lessons learned from remote and harsh environment deployments can be applied to create more robust and scalable solutions for assessing indoor radon risk, particularly in underserved, resource-limited settings.

## 4. IoT Radon Sensing Technologies: Principles, Components, and Trade-Offs

Effective radon mitigation depends on timely and accurate environmental data, which traditional radon monitoring systems rarely provide at scale. IoT technologies offer real-time, distributed sensing with high spatial and temporal resolution [13,15]. Radon, being a highly variable indoor air contaminant, requires continuous, high-resolution data capture to allow meaningful risk mitigation. The design of IoT-based radon monitoring systems encompasses several interconnected areas, including sensor selection, embedded hardware setup, communication infrastructure, and data quality assurance. Each design decision balances sensitivity, scalability, cost, and energy efficiency [20,29]. The following subsection outlines the main technical components of IoT radon systems with reference to recent developments and validated studies.

### 4.1. Radon Detection Methods for Embedded Systems

**Figure 2:**
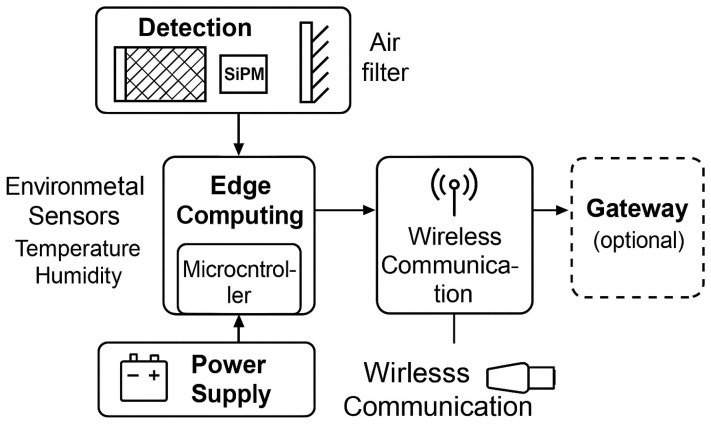
Schematic of an IoT radon sensor node showing the key system components: radon detection module (SiPM, PIN photodiode, or MOS sensor), environmental sensors (e.g., temperature, humidity), microcontroller for edge computing, wireless communication module (e.g., LoRaWAN, NB-IoT, Wi-Fi), and power supply. Data is processed locally and transmitted wirelessly, optionally through a gateway, to remote cloud or edge analytics systems.

The sensing mechanism used in an IoT radon detector is key to its performance, as it affects the device’s sensitivity, response time, energy consumption, and autonomy. One of the most common technologies is the PIN photodiode sensor, which works by collecting radon progeny electrostatically and detecting alpha particles through a solid-state semiconductor [20]. These sensors are popular in low-power designs because of their lower intrinsic sensitivity and vulnerability to environmental noise, which require longer integration times and careful signal processing to ensure accurate data [30]. A more advanced option is the use of silicon photomultipliers (SiPMs, Figure 2) combined with scintillators. In this setup, alpha particles from radon decay events cause light emissions within a scintillating medium, which are then amplified and measured by the SiPM. This method offers a significant improvement in detection sensitivity and timing accuracy [31]. SiPM-scintillator arrays have proven particularly useful in research applications and volcano monitoring, where both sensitivity and durability are essential [32]. However, their higher power consumption and need for analog signal processing circuits can make integration into long-lasting, battery-powered embedded systems more challenging. Another category of radon sensors used in IoT platforms relies on metal-oxide-semiconductor (MOS) technologies, such as those found in commercial devices like RadonEye. These sensors operate by detecting changes in conductivity resulting from radon-induced ionization in the surrounding air. While they are cost-effective and provide immediate reading, they tend to experience calibration drift over time and are sensitive to humidity, temperature, and other interfering gases [33]. Consequently, deploying them in critical infrastructure requires ongoing recalibration or the use of software-based drift correction models [34]. Recent innovations have explored the use of microelectromechanical systems (MEMS) and nanomaterials, such as graphene-based composites, to develop radon detectors that combine miniaturization with enhanced sensitivity [35,36,37]. Although these technologies are still primarily in the experimental stage, their potential to alter IoT radon sensing through ultra-low-power operation and high spatial resolution is significant and warrants further research. The core hardware structure of a typical IoT radon sensor node is illustrated in Figure 1, which depicts the modular design, including sensing, environmental compensation, edge processing, wireless communication, and power supply.

### 4.2. Embedded System Design

The hardware architecture of an IoT radon sensor is centered around its embedded computing platform, which manages sensor interfacing, data processing, communication scheduling, and power regulation. The selection of a microcontroller is largely dictated by the specific deployment environment and the connectivity requirements of the application. For instance, the ESP32 platform is a popular option for indoor or localized monitoring due to its dual-core processor and integrated Wi-Fi and Bluetooth Low Energy (BLE) modules [38]. This enables smooth integration with home or institutional networks and supports real-time data streaming at a relatively low cost [39]. In contrast, for large-scale or infrastructure-poor environments, microcontrollers embedded with LoRa transceivers such as STM32 paired with SX1276 chips are widely adopted [40]. These systems benefit from long-range, ultra-low-power communication and can operate for months or even years on battery power alone. The embedded firmware in such systems often implements aggressive power-saving strategies, including deep sleep modes, timed wake cycles, and event-driven interrupts to minimize energy consumption without sacrificing measurement reliability [41]. Power autonomy is a key constraint. Most systems use lithium-ion battery pack sized to match duty cycles, while some employ solar or thermoelectric generators to extend operating life [42]. In some deployments, solar panels or thermoelectric generators are used to recharge onboard batteries, significantly extending the operational lifespan. Efficient voltage regulation, low-leakage circuits, and hardware watchdog timers are additional techniques used to ensure uninterrupted data collection over extended periods [43,44].

### 4.3. Connectivity Architectures

**Figure 3:**
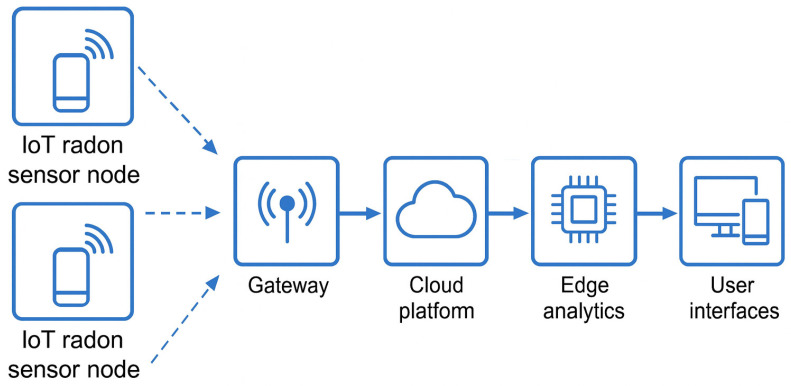
System-level architecture of an IoT-based radon monitoring system. Sensor nodes measure radon levels and send data wirelessly to a central gateway, which then sends it to a cloud platform. The edge analytics detect trends, trigger alerts, and remove false readings. Users interact with the system through web or mobile interfaces that display real-time data, thresholds, and alerts.

A key feature of IoT systems is their ability to transmit data wirelessly; the choice of communication protocol significantly affects network scalability, energy efficiency, and deployment costs [45]. Several wireless technologies are currently employed in modern IoT networks, each offering distinct benefits and limitations, depending on the specific application. LoRaWAN is the most common protocol for long-range, low-power use cases. It provides reliable connectivity over distances up to 15 km while consuming minimal energy, making it ideal for rural deployments or dense sensor networks in urban monitoring systems [46,47,48,49]. Its effectiveness has been shown in various environmental sensing scenarios, including volcanic radon monitoring and industrial safety networks [50,51]. However, LoRaWAN’s bandwidth limitations and duty-cycle regulations restrict its use in scenarios requiring high frequency or large data volumes [52]. NB-IoT presents a strong alternative in urban areas with existing cellular infrastructure. Operating on licensed LTE spectrum, it delivers excellent indoor coverage and reliable communication even inside heavily obstructed buildings [53]. Although it consumes more energy than LoRaWAN, its high-quality service and carrier-grade reliability make it suitable for public infrastructure and smart city initiatives [14,54]. Other short-range protocols, such as Wi-Fi and BLE, work well in localized settings where power resources are less limited and network access is readily available. These technologies support high-bandwidth data transmission and are compatible with consumer devices, enabling real-time visualization and user interaction. However, their limited range and higher energy consumption restrict their use to specific applications such as home monitoring or educational installations. Table 3 offers a comparative overview of these technologies, outlining their operating parameters and suitability for radon monitoring. Figure 3 summarizes the overall system architecture of a typical IoT-based radon monitoring network, illustrating how data flows from sensor nodes through wireless transmission to cloud platforms, where analytics and visualization occur.

### 4.4. Calibration and Validation Requirements

Ensuring the accuracy and reliability of IoT radon sensors requires rigorous calibration and validation methods that align with national and international standards. Calibration involves establishing the link between sensor output and actual radon levels, usually expressed in becquerels per cubic meter (Bq/m^3^). This is typically done through laboratory systems, where sensors are exposed to controlled radon atmospheres generated from known radioactive sources, such as ^226^Ra. Calibration facilities typically operate under the traceability standards established by the National Institute of Standards and Technology (NIST) in the United States or the Physikalisch-Technische Bundesanstalt (PTB) in Germany [79,80]. Xu et al. [81] demonstrated a closed-loop air circulation system that achieved calibration reproducibility of ±2% using precisely characterized radon sources and cross-comparison with commercial RAD7 detectors. Such precision is essential for ensuring consistency between devices in multi-node sensor networks. Complementing laboratory calibration, field validation is carried out by collocating IoT sensors with regulatory-grade monitors, such as AlphaGuard (Bertin Technologies, Aix-en-Provence, France) or RAD7 units, in real-world settings [82,83]. This process evaluates performance under environmental stressors, such as temperature fluctuations, humidity, and atmospheric pressure, which can impact sensor response. Emerging methods are tackling the logistical and safety issues of traditional calibration. For example, Ren and Liu [24] developed a new approach that eliminates the use of radioactive calibration sources by employing standard water samples with known concentrations, thereby enabling safe and affordable sensor validation in seismic observation systems. Ongoing accuracy maintenance is essential. Many modern IoT radon devices include software-based drift compensation algorithms that adjust for sensor aging and environmental interference using statistical or machine learning models. This helps ensure that long-term monitoring data stays useful without needing frequent physical recalibration, which is especially important in large-scale or hard-to-reach deployments. Among the five reviewed studies, only two provided detailed calibration outcomes. Yousefian et al. [17] validated their prototype against AlphaGuard (Bertin Technologies, Aix-en-Provence, France) in laboratory settings, demonstrating measurement consistency within ±8% of regulatory standards. The remaining studies either relied on trend validation [13] or user feedback [18], highlighting the need for more robust, standardized calibration protocols.

### 4.5. In-Depth Exploration of Sensor Technology

The performance and reliability of IoT-based radon monitoring systems mainly rely on their core sensing technology. While radon-specific detectors remain limited, valuable insights can be gained from existing detection methods and innovations in the wider IoT environmental sensing field. These combined approaches reveal trade-offs among sensitivity, power use, durability, and cost, which impact the practicality of large-scale radon monitoring. PIN photodiodes are among the most frequently used devices in radon IoT systems because they detect alpha particles from radon progeny via semiconductor junctions. Their popularity is due to their compact size, low power consumption, and compatibility with embedded platforms. However, these benefits come with reduced intrinsic sensitivity, which necessitates longer integration times and careful signal processing to reduce environmental noise. Consequently, PIN photodiodes are well-suited for continuous, though somewhat coarse, monitoring—particularly in residential or institutional environments where energy efficiency and cost are key factors [30]. A more advanced category of detectors integrates scintillators with silicon photomultipliers (SiPMs). In these setups, alpha particles hitting a scintillating material generate light, which is then amplified and detected by SiPMs. This method provides significantly greater sensitivity and faster response times compared to solid-state diodes, making it particularly advantageous for research applications like monitoring volcanic emissions or seismic activity, where short-term fluctuations are critical [31]. However, the complexity of analog signal processing and increased power pose challenges for sustained, battery-powered IoT deployments.

Commercial radon detectors and many prototype IoT platforms use metal oxide semiconductor (MOS) sensors, which identify shifts in conductivity triggered by ionizing radiation in the air. These sensors are affordable, deliver near-instant readings, and are ideal for consumer devices. However, their accuracy can decline over time due to baseline drift influenced by temperature, humidity, and interfering gases. While recalibration can counteract this, it poses practical challenges for large-scale distributed systems. Recent developments in environmental IoT aim to solve this by employing software algorithms, often based on statistical or machine learning techniques, to correct for drift and ensure long-term reliability without frequent manual recalibration [33,34]. Looking to the future, the use of microelectromechanical systems (MEMS) and nanomaterials presents an exciting opportunity. Researchers are exploring new composites like graphene-based films and nanostructured metal oxides for their distinctive electrical and chemical traits, which provide ultra-high sensitivity, quick response times, and low power consumption [36]. While these technologies remain largely experimental, they closely match the requirements of IoT-enabled radon monitoring, such as miniaturization, energy efficiency, and dense deployment capability. If these sensors can be scaled successfully, they could enable ongoing radon monitoring in areas that now lack such capabilities due to power limitations. Beyond the sensing materials themselves, wider trends in IoT design also affect radon monitoring. Reviews of low-power environmental sensors emphasize the move toward embedding computational capabilities within the sensing node, enabling basic signal processing, noise filtering, and anomaly detection to occur before data is transmitted [82]. This edge intelligence decreases communication demands, prolongs battery life, and ensures only the most pertinent data reaches the cloud. Likewise, advances in energy harvesting, such as piezoelectric and thermoelectric generators, allow sensors to generate power from ambient vibrations, temperature differences, or light. Recent research indicates that combining harvesters with IoT nodes can increase their lifespan by up to nine times, which is highly relevant for radon systems placed in remote or hard-to-access areas [84]. Current IoT radon systems mainly use PIN photodiodes or MOS detectors, but further designs are likely to combine sensitivity of scintillator-SiPM systems with the efficiency of MEMS and nanomaterials. Advances in edge processing and energy harvesting may also reduce calibration drift and extend battery life. Viewing radon sensing within the larger development of IoT sensor technology shows that innovations in air quality, industrial safety, and environmental hazard detection can accelerate progress in radon IoT solutions, leading to more reliable, affordable, and accessible exposure assessments.

### 4.6. Wider Uses of Environmental IoT Sensors-Similar Lessons

While research directly combining radon monitoring with IoT remains scarce, a broader range of IoT-based environmental sensing studies offers valuable insights. Fields like air quality, agriculture, industrial safety, and hazard monitoring encounter similar challenges, such as calibration drift, sensor placement, power efficiency, data accuracy, and deployment logistics. Studying these related areas helps extract lessons that can be applied to develop better radon-specific IoT solutions more quickly. Indoor air quality networks developed over the past decade provide useful models. Low-cost IoT devices, including microcontrollers like the ESP32, have been effectively used to track carbon dioxide, particulate matter, and volatile organic compounds in real time in settings such as schools, universities, and offices [85]. These systems generally combine multiple sensors on a single unit, send data wirelessly to cloud servers, and present results via intuitive dashboards. Although they do not measure radon directly, they show that continuous indoor exposure monitoring is feasible at a scale and cost previously thought impossible. The insights gained from designing these networks, especially regarding calibration, user accessibility, and data visualization, offer a valuable blueprint for developing similar radon monitoring systems in homes and educational facilities.

Agricultural and industrial IoT systems face similar challenges and offer relevant comparisons. In precision farming, networks of soil moisture, temperature, and gas sensors face similar challenges, such as intermittent connectivity, energy limitations, and the need for durable enclosures to handle changing environments. In the mining and petrochemical sectors, IoT methane and carbon monoxide monitors are designed with a focus on worker safety, incorporating low-latency alerts, sensor redundancy, and autonomous battery systems. These design principles align with the needs of radon monitoring, particularly in workplaces where quick detection of high radon levels is critical and reliable operation in tough conditions is essential. Large-scale hazard monitoring highlights the versatility of IoT technologies. A notable example is using distributed sensor networks for volcanic gas monitoring, where LoRaWAN-enabled nodes have been placed on Mount Etna to measure radon and carbon dioxide in difficult environments [86]. These networks are built to operate in remote, low-power settings, transmitting data over long distances with minimal infrastructure. The approaches used in volcanology—such as sensor robust encapsulation, duty cycling to save power, and adaptive data transmission—are highly relevant for expanding radon IoT networks into rural or underserved regions.

The integration of artificial intelligence and machine learning into environmental IoT towards more sophisticated radon monitoring systems. Recent reviews indicate that AI-enhanced IoT architectures can better detect anomalies, forecast pollutant trends, and adjust for sensor drift in real time, resulting in more reliable and actionable data [22]. These methods could be applied to radon detection, especially in cases where low-cost sensors might otherwise be overlooked due to concerns about accuracy. By incorporating computational intelligence into IoT platforms, radon monitoring networks can deliver not just raw concentration measurements but also predictive insights on exposure risks across varying environmental conditions. Collectively these examples show that the technical challenges in radon IoT research mirror those in other fields and can be addressed though similar solutions. Technical challenges such as calibration, power management, and large-scale deployment have been addressed in related fields, with solutions like hybrid sensor arrays, energy harvesting, and AI-based correction models readily adaptable for radon. Although this section emphasizes thematic and technological parallels across environmental monitoring sectors, it is equally vital to consider specific real-world examples of how these principles have been implemented.

## 5. Deployment Strategies: Indoor Mapping to Large-Scale Networks

Effective integrating IoT-based radon sensors into environmental health systems requires deployment strategies tailored to the spatial, temporal, and social factors. Low-cost, real-time monitoring is valuable, but its public health impact depends on practical deployment. This section examines spatial mapping, temporal patterns, and deployment scales, drawing on research and case studies.

### 5.1. Mapping the Spatial Distribution of Radon

Radon infiltration and buildup vary significantly across the vertical and horizontal dimensions of built environments, mainly due to the interaction of geology, construction methods, and ventilation design. Multi-node sensor arrays enable high-resolution spatial mapping, which helps identifying anomalies linked to substructures and supporting targeted mitigation. Radon levels are often highest in subgrade structures, such as basements and crawl spaces, due to direct contact with soil gas and limited airflow [87]. In a Macedonian study involving 76 schools and homes, basement radon concentrations were found to be up to 15 times higher than those on the ground floor [88]. IoT systems using this zoning approach can pinpoint microenvironments within a building that pose disproportionate health risks, enabling targeted mitigation such as localized sub-slab depressurization or room-specific ventilation updates. Building materials also affect radon variability. Granite and phosphate-based materials used in flooring and countertops tend to emit radon, especially when combined with airtight building envelopes that restrict natural ventilation [89,90]. Deploying sensors across different floor levels and material zones supports building-specific risk models rather than reliance on general thresholds. Real-time mapping integrated with WebGIS platforms (e.g., RnMonitor), improves detection, visualization and accessibility, encouraging broader engagement in mitigation [13].

### 5.2. Temporal Dynamics and Environmental Drivers

Radon is a dynamic contaminant whose concentration fluctuates in response to daily, seasonal, and weather-related factors. Capturing these fluctuations is essential for accurate exposure assessment and the development of effective mitigation systems. Unlike passive detectors, IoT systems provide continuous data, enabling detection of transient peaks that may be hidden in average data. Recent research, utilizing 13 years of data from 46 U.S. states, confirms that indoor radon levels exhibit strong seasonal patterns, with peaks in January and February attributed to reduced ventilation and thermal stacking effects [23]. Diurnal cycles cause nighttime elevations, driven by negative pressure differences that draw radon inside from the ground during times of decreased HVAC activity [91]. Environmental triggers, such as storms, drops in barometric pressure, and soil saturation from rainfall, are now systematically incorporated into predictive analytics platforms, with radon sensors interfacing with external weather APIs [92]. Similarly, human behavior—especially HVAC use and window opening—has been quantified in classrooms, where radon levels decreased by over 50% after behavioral changes prompted by IoT alerts [18]. Statistical models and correction algorithms have been developed to convert short-term IoT data into reliable estimates of long-term exposure, accounting for seasonal biases and building-specific factors. New correction tools, such as the coefficient of temporal variation K(t), are being proposed as standardized measures for cross-study comparisons [19,93].

### 5.3. Real-World Deployment Scales

Deployment at scales ranging from single buildings to communities presents opportunities and challenges for effectiveness of radon exposure assessment. These deployment strategies must be tailored to the infrastructural, regulatory, and behavioral realities of the specific context in which they are implemented. On the scale of individual buildings, IoT radon sensors are increasingly used in smart homes, residences, schools, and workplaces. These systems often include embedded microcontrollers like the ESP32, which offer Wi-Fi or Bluetooth Low Energy (BLE) connectivity to support real-time data transfer and device control. In residential and institutional settings, where power and connectivity are readily available, these systems enable continuous radon monitoring and quick responses. For example, in educational environments, radon sensors have been deployed to trigger ventilation adjustments during periods of exposure, leading to substantial reductions in radon levels. The school AIR framework in Portugal, for instance, used low-cost sensors in classrooms to monitor indoor air quality, enable real-time ventilation adjustments, and raise awareness among students and staff [18].

On larger scales, community-wide networks have been set up to monitor radon levels across multiple buildings in both urban and rural areas. These systems often use long-range, low-power communication protocols such as LoRaWAN or NB-IoT, which are essential for maintaining reliable connectivity in infrastructure-limited settings [16]. A notable example is Tehran, where an IoT-based network of 120 radon sensors was installed in public schools. This network identified persistent hotspots, especially in basement classrooms that exceeded international safety standards, ultimately influencing local policy actions and funding for remediation [17]. The use of geospatial platforms like RnMonitor has further enhanced the effectiveness of these monitoring networks. This WebGIS-based system allows hierarchical organization of sensor nodes across rooms, buildings, and districts, providing a spatially aware view of radon exposure and facilitating centralized risk analysis [13]. These systems are particularly valuable for regional health agencies aiming to prioritize cleanup efforts in high-risk areas and ensure compliance with national radon regulations. Citizen science projects using low-cost radon kits show potential to enable public participation in environmental data collection but face challenges with data integrity. These efforts have demonstrated the feasibility of using distributed, non-expert networks to generate valuable databases while also increasing public awareness and engagement. However, they face significant challenges in ensuring data integrity. Issues such as sensor drift, improper placement, lack of calibration, and inconsistent metadata can weaken the scientific usefulness of the data. To address these challenges, studies have proposed quality control strategies including periodic validation against regulatory-grade instruments and the use of automated anomaly detection algorithms [19,94].

Table 4 summarizes major deployment case studies, highlighting insights and limitations across different scales. In Alberta, Canada, a network of 50 nodes tracked HVAC-radon dynamics during winter, while in Seoul, Republic of Korea, a year-long deployment in 200 buildings revealed how architecture and materials affect exposure levels. These case studies demonstrate the scalability and flexibility of IoT radon systems and emphasize the need to customize deployment strategies based on the sociotechnical conditions of each site.

Deployment—whether in a single building or across an entire municipality—must consider operational constraints, stakeholder involvement, data handling, and sustainability. Integration into public health systems will require technical improvements, cross-sector collaboration, and regulatory support. Linking sensing with participatory governance can shift radon risk management from a reactive to proactive.

### 5.4. Lessons from Other IoT Domains Applied to Radon Monitoring

Real-world IoT deployments in other domains provide practical lessons from radon monitoring. Case studies from volcanology, classroom air quality, and industrial gas sensing illustrate solutions to challenges such as ruggedization, low-power operation, remote connectivity, and real-time alerts. These examples inform the design of scalable, reliable radon-specific IoT networks. A notable similarity exists in volcanic gas monitoring, where radon is one of the key gases tracked in extreme conditions. For example, on Mount Etna in 2019, a LoRaWAN sensor network was set up to monitor radon variations linked to volcanic activity [86]. This system employed rugged housing, long-range wireless protocols, and duty cycling to conserve power in challenging environments. Although designed for volcanology, these apply to remote homes, rural schools, or underground workplaces with limited infrastructure and high monitoring needs. In indoor environments, the School AIR project in Portugal deployed low-cost CO_2_ sensors in classrooms to support ventilation decisions and improve air quality. This model is highly transferable to radon, especially in schools and childcare settings. For example, Barros et al. pilot radon monitoring initiative in Boston classrooms led to behavioral changes that significantly reduce radon exposure, mirroring the impact seen with CO_2_-based interventions. Industrial safety systems provide additional insights. In mining and oil industries, IoT-based methane and carbon monoxide detectors support real-time alerts and redundancy in high-risk zones. These priorities, low latency detection, minimal false alarms, and offline functionality, closely align with radon monitoring requirements in occupational settings such as underground facilities or older public buildings. facilities or older public buildings. Applying these tested approaches can accelerate the development of robust radon-specific IoT solutions, moving beyond prototypes and into public health practice.

### 5.5. Examples of Real-World Deployments

Demonstrating that IoT-based radon monitoring works in real-world settings is vital for understanding how these technologies can support large-scale mitigation efforts. Several recent case studies have shown successful deployments in various environments, including schools, homes, workplaces, and community health programs. One example in public buildings is the RnMonitor platform, developed and piloted in Portugal. This system combines commercially available radon detectors with a WebGIS interface and a LoRaWAN communication backbone. By deploying it across municipal buildings and schools, RnMonitor allows for real-time, room-level indoor radon monitoring [13]. Building on this research, a major study of radon levels in 533 schools across the UK highlighted the need for detailed monitoring. The study showed that radon levels can differ greatly within a single building, even from one room to another. These findings emphasize the importance of using dense sensor networks that can provide localized data. IoT systems are well-positioned to support this type of distributed sensing and real-time data aggregation [95].

Residential environments are another key area for deployment. A 291 day study in a domestic basement compared the Radon Eye with a laboratory-grade AlphaGUARD detector and found that it consistently and reliably measured radon levels in real-world settings, making it a cost-effective alternative for long-term home monitoring. These results are especially important for public health campaigns targeting radon exposure in underserved or rural communities, where affordability and ease of use are crucial [96].

Public health programs have shown that distributed radon monitoring is scalable. A nationwide project in Northamptonshire, United Kingdom, deployed over 1000 electret-based radon detectors in residential properties to evaluate exposure risks. The initiative highlighted both cost-effectiveness and logistical feasibility, demonstrating how large-scale monitoring can be implemented using low-maintenance technologies. This campaign served as a model for incorporating radon risk assessment into broader community health strategies [97]. Lastly, research on radon levels in homes and workplaces highlights the benefits of comprehensive monitoring approaches. A study in Los Alamos County, New Mexico, installed passive track-etch detectors in both office spaces and homes. The findings showed that residential radon exposure levels were, on average, up to eight times higher than those in workplaces. This emphasizes the need to include private residences in radon risk assessments and suggests the importance of integrated monitoring frameworks that cover both workplace and residential environments [98]. Table 5 summarizes the key features of the case studies discussed, including the technologies used, validation results, and broader implications for scalable radon monitoring. This comparison highlights how IoT-based systems can adapt to various environmental, institutional, and community contexts.

## 6. Data Lifecycle Management: Edge to Cloud to Insight

Maximizing public health value of IoT-enabled radon sensors requires converting raw data into accurate, timely, and actionable insights. This requires a complete data lifecycle that includes edge signal processing, cloud infrastructure, real-time visualization, and epidemiology-relevant exposure metrics. Well-managed data flows support mitigation strategies, guide long-term policy design, and reduce health risks.

### 6.1. Edge Processing and Compression

Edge computing reduces latency, conserves energy, and ensures that only relevant data is transmitted [99]. Embedded radar sensors often use on-device averaging algorithms to smooth out stochastic fluctuations, especially in environments with HVAC cycling or human activity that create signal noise. Outlier rejection methods, such as moving z-score or IQR filters, exclude false peaks caused by electromagnetic or mechanical interference. Advanced platforms also apply Fast Fourier Transform (FFT) filtering to detect and suppress frequency-domain noise patterns. Spectral processing improves detection reliability without heavy computational demands, an essential feature for battery-powered microcontrollers like the STM32 or ESP32 [100]. Supporting this, RnProbe, a LoRa-enabled edge device, uses local logic to decide when radon levels exceed thresholds, prompting higher sampling rates or immediate data transmission [15]. Energy-efficient scheduling further extends device lifespan. Studies show that edge filtering and batch transmission can reduce cloud data loads by more than 50% and extend battery life by 130%, a critical advantage in schools, remote homes and off-grid infrastructure [101]. These efficiencies are especially critical in deployments across schools, remote homes, and essential infrastructure with limited grid access.

### 6.2. Cloud Infrastructure and Storage

Once data is transmitted, the cloud serves as the central hub for long-term storage, integration, and advanced analytics. Major platforms such as AWS IoT Core and Azure IoT Hub provide managed pipelines that enable seamless device provisioning, secure MQTT/HTTPS protocols, and scalable data lakes for processing. AWS supports integration with Amazon Timestream for real-time time-series analytics, while Azure excels in Power BI dashboards and digital twin modeling of built environments. For open-access or low-budget deployments, ThingsBoard offers a robust open-source alternative that supports rule-based processing, device telemetry, and third-party integration without vendor lock-in [101]. From a storage perspective, InfluxDB, a high-throughput time-series database, performs well in managing large volumes of sensor readings with millisecond resolution and supports down sampling, retention, and continuous queries. This structure supports detailed analysis of diurnal and seasonal trends. PostgreSQL and its spatial extension PostGIS offer greater flexibility for joining environmental radon data with building metadata, GIS overlays, or health records, supporting spatial epidemiology and exposure zoning [102].

### 6.3. Visualization, Alerts and User Interfaces

Visualization translates raw data into accessible risk information. Platforms like Grafana and Ubidots provide customizable dashboards that allow stakeholders to monitor radon levels in real-time, identify spatial hotspots, and analyze long-term exposure patterns. Visual tools are essential not only for public engagement and operational responses in schools, hospitals, and homes [103]. Alerts can be configured via SMS, email or mobile apps when radon levels exceed 100 Bq/m^3^ or other regulatory limits. Alerts can also be linked to environmental triggers like low barometric pressure or specific times, such as during school hours, enabling adaptive HVAC adjustments or ventilation responses. Recent systems like RnMonitor have combined sensor data with WebGIS platforms to support spatial risk communication, helping health officials prioritize interventions across cities, districts, or vulnerable areas [15].

### 6.4. Exposure Metrics and Risk Modeling

Public Health interpretations require data to be translated into metrics and models. The Time-Weighted Average (TWA) remains a standard measure, but newer models add occupancy-adjusted exposure estimates, weighting radon levels by human presence, particularly in settings like schools and elder care centers. Combining environmental data with epidemiological models can generate personalized or population-wide risk assessments. For instance, models that incorporate indoor radon exposure along with factors like age, smoking history, and building type have shown predictive value for lung cancer risk [104,105,106]. Other studies suggest using multiple regression models to analyze how environmental factors such as humidity, temperature, and pressure influence radon levels, resulting in more accurate, site-specific exposure predictions [107]. Machine learning models are now integrated platforms using historical data to forecast peaks or detect sensor drift. These tools improve anomaly detection and seasonal adjustment into long-term exposure risk estimates.

## 7. Critical Challenges: Barriers to Real-World Impact

Despite advances in edge-to-cloud radon monitoring, their practical use is still limited by environmental interference, calibration difficulties, and socio-political factors. Addressing these challenges is essential if IoT radon sensing is to deliver public health benefits.

### 7.1. The Validation Crisis

A central difficulty in deploying distributed IoT radon systems is achieving reliable measurements in real-world conditions. While most sensors are calibrated in controlled laboratory environments, actual field settings with fluctuations in humidity, temperature, and pressure often lead to signal drift, non-linearity, and decreased accuracy. Emirhan [20] found that even well-designed active detectors can give variable results when ambient humidity and temperature change, highlighting the delicate connection between environmental factors and sensor reliability. Standardization remains inadequate for modern real-time devices. ISO 11665-9, designed for passive or manually operated devices, does not account for the telemetry, sampling frequency, and on-device processing that are now common in smart radon monitors. Without updating validation protocols, maintaining cross-comparability and regulatory compliance remain challenging [11]. Large scale calibration at scale is logistically difficult challenges. Achieving precision across varied locations demands scalable approaches such as periodic reference checks, mobile calibration units, or AI adjustments models [108].

### 7.2. Power and Connectivity Constraints

Edge-based monitoring depends on balancing with low energy use. Hourly measurements can drain batteries quickly, and large-scale deployments can be required frequently, which is often impractical in areas like crawl spaces or rural homes [109]. Connectivity is another major constraint. Basement installations often suffer from weak signals due to thick walls and underground placement. Although LoRaWAN provides long-range coverage with low power use, it has limited data capacity and known vulnerabilities, making it less effective for urgent alerts or large data loads [15]. The RuraTHINGS project showed that even with hardware tuned for low power, backhaul connectivity issues and latency limit real-time response [109].

### 7.3. Security, Privacy and Ethics

Deploying radon sensors in private homes raises ethical concerns, primarily regarding privacy and legal implications. These sensors that track indoor conditions like air exchange, occupancy, or room use can accidentally share private behavioral details. These concerns are reflected in laws such as GDPR and CCPA, which mandate clear consent, limited data collection, and strict purpose use. Despite the sensitivity of data, IoT communication protocols like LoRa and MQTT are not always equipped with full-stack encryption or secure identity management. Without strong authentication and encrypted storage, these systems remain vulnerable to breaches, spoofing, or data manipulation [21]. Ethical frameworks for consent and governance are still evolving. In shared or rented housing, the ownership of data and access rights remains unclear reducing trust and limited adoption. This ambiguity can erode public trust and hinder adoption [110].

### 7.4. Socio-Economic Equity and Accessibility

Smart radon sensing currently benefits mainly urban, digitally literate populations while risks are concentrated among digitally literate, urban populations, leaving behind rural, elderly, or low-income households that often face the highest exposure. IoT radon monitors cost more than $50 per unit, well above charcoal kits, and often require mobile connectivity and digital interfaces. Studies such as those by Khan et al. [111] show that low-cost interventions are more acceptable in vulnerable populations who may lack both financial resources and digital literacy to interpret sensor feedback effectively. Rural connectivity efforts further widen the digital divide. Even where sensors are deployed, weak backhaul infrastructure hinders real-time communication. Ladeira et al. [109] observed that residents in such regions often could not act due to a lack of awareness, technical support, or adequate mitigation infrastructure.

## 8. Future Directions

Radon remains a significant but preventable health risk. Advances in sensors, artificial intelligence, and adaptive infrastructure create opportunities to shift monitoring from a passive, fragmented practice into an active, intelligent, and equitable public health tool. This section presents a future-oriented roadmap derived from the challenges and opportunities highlighted in the review.

### 8.1. Next-Generation Sensor Technologies

Recent progress in material science, sensor miniaturization, and low-power electronics has expanded opportunities for scalable environmental monitoring. Technologies such as MEMS-based detectors, graphene-coated transducers, and hybrid nanomaterials are increasingly employed to detect gases, including carbon dioxide, methane, and volatile organic compounds. These sensors provide enhanced sensitivity and reduced power use, supporting distributed, wireless monitoring over long durations [112,113,114]. Radon detection presents distinct challenges. Unlike gases measured via electrochemical or infrared techniques, radon is a radioactive noble gas that needs detection of alpha particles or its decay products. Common methods include alpha particle spectroscopy, scintillation, or indirect detection with ionization chambers and MOS sensors. These techniques are more susceptible to environmental factors such as humidity and temperature, which can influence the stability and accuracy of measurements over time [115,116]. In practical deployments like the Tehran school monitoring network, MOS sensors demonstrated potential for low-cost deployment but were influenced by humidity-related drift. Automatic calibration based on environmental conditions could improve long-term performance and reduce maintenance needs [17]. This is especially key for networks in schools, homes, or rural areas where regular maintenance might be limited. Miniaturized alpha detectors and electrostatic collection systems are emerging, designed for wireless integration. They aim to deliver precise and energy-efficient radon measurements in small formats, suitable for large-scale IoT applications [117]. Future sensor deployments must address calibration stability, environmental resilience, and energy efficiency to enable reliable large scale public health monitoring.

### 8.2. Data Analytics and Predictive Monitoring

As radon monitoring networks grow, interpreting and responding to large data sets becomes ever more important. Besides providing real-time measurements, data analytics can enhance system reliability, reveal patterns in radon exposure, and enable early interventions. For instance, analyzing sensor data over time can help detect malfunctions, calibration issues, or data gaps, as seen in pilot projects like the Boston classroom monitoring initiative, where uncalibrated sensors led to gaps in long-term exposure assessment. Predictive modeling is also useful for planning radon mitigation. In Tehran, continuous monitoring of 120 schools showed seasonal radon patterns, with peaks in winter when HVAC systems were less active [17]. This data can help predict high-risk periods and inform ventilation scheduling or specific mitigation efforts before levels surpass safe thresholds. Long-term analytics can reveal high-risk buildings or rooms by analyzing exposure data over time, beyond seasonal trends. This enables health authorities or school administrators to focus mitigation efforts on areas with real exposure patterns, rather than relying on fixed building characteristics. As radon sensing becomes more common, integrating essential analytical features like alert thresholds, trend detection, or exposure tracking into the monitoring system could greatly improve its effectiveness for public health, especially in areas with limited professional oversight [118].

### 8.3. Mitigation Integration and Automation

Next-generation radon management systems are expected to integrate both monitoring and active mitigation. Closed-loop systems, connect sensors directly to building infrastructure such as HVAC units, exhaust fans, or Energy Recovery Ventilator (ERV) systems to adjust airflow based on real-time radon levels automatically [119,120,121]. These systems provide continuous protection with minimal user intervention and are especially valuable in institutional settings and healthcare facilities. Blockchain-based frameworks are being explored to improve the accuracy and traceability of mitigation records. By logging radon levels and mitigation events in tamper-proof records, blockchain technology can support regulatory audits, insurance compliance, and transparency between tenants and landlords [122]. Additionally, this technology opens new opportunities for incentive programs linked to verified environmental performance, such as tax credits or health insurance benefits.

### 8.4. Policy, Standardization and Scalable Adoption

Regulatory frameworks must evolve to mandate continuous monitoring rather than static, annual average monitoring. Continuous data streams offer superior temporal resolution and responsiveness, which are essential for modern risk modeling and real-time mitigation. Yet current guidelines, such as the ISO 11665-9 standard, are ill-equipped to validate dynamic, telemetry-driven systems [10]. Adoption can be accelerated through open-source hardware and software platforms that support low-cost, customizable, and transparent radon monitoring. Citizen science initiatives that leverage such platforms have already shown their usefulness in data collection and public engagement. However, ensuring the scientific reliability of these systems will require built-in calibration protocols, metadata standardization, and periodic validation checks [123]. Public–private partnerships are essential for deploying these technologies in high-risk, under-resourced communities. Government-backed subsidies for IoT sensors, in collaboration with technology firms and housing authorities, can enable equitable access to radon risk reduction. These partnerships can also fund training programs to build local capacity for sensor installation, data interpretation, and responsive mitigation.

## 9. Conclusions

This review has examined the emerging field of IoT-enabled radon monitoring, focusing on sensor technologies, system designs, deployment strategies, and data workflows. Despite an extensive search, only five key studies meet the inclusion criteria, highlighting that this area is still in its infancy. Rather than indicating a limitation, this small evidence base reflects the novelty of the research and underscores the urgent need for further investigation. By placing radon sensing within the wider context of environmental IoT applications like air quality, industrial safety, and volcanic gas monitoring, this review shows that many challenges, such as calibration drift, power autonomy, and scalability, have already been addressed in related fields. Insights from these domains, along with advances in sensor development, energy harvesting, and AI analytics, provide clear paths for creating robust, affordable, and scalable radon IoT solutions. By aggregating initial efforts and linking them to broader technological trends, this review sets baseline references and outlines a future research agenda. With ongoing innovation and interdisciplinary efforts, IoT-based radon monitoring has the potential to shift management from static, reactive assessments to proactive, real-time public health protection.

## Figures and Tables

**Table 1:** Comparative summary of foundational IoT-Based radon monitoring studies.

Study	Sensor Type	Deployment Context	Connectivity	Calibration & Validation	Power Configurations	Key Findings	Reported Limitations
[17]	Metal-oxide Semiconductor (MOS)	120 public schools (Tehran)	NB-IoT	Co-location with RAD7; baseline environmental adjustment	Battery-operated with low-power duty cycling	Persistent radon in basement classrooms; informed local remediation policy	Calibration inconsistencies; variable device responses across humidity levels.
[19]	Active detectors	50 residential buildings (Alberta, Canada)	Wi-Fi	Cross-referenced with HVAC telemetry and seasonal indoor-outdoor trends	Grid-connected indoor devices	Radon peaks correlated with winter heating cycles and HVAC operations	Limited study duration (6 months); lack of standardized calibration
[13]	Commercial Sensor array with WebGIS integration	Government and institutional buildings (Portugal)	Wi-Fi + Web-based GIS	Trend comparison with reference instruments; visual validation	Mains power; no battery autonomy	Enabled real-time spatial mapping of radon levels; facilitated risk communication	Limited deployment scale; dependent on fixed power infrastructure
[18]	Open-source consumer-grade sensors	30 school classrooms (Boston, USA)	Wi-Fi and BLE	Software-based calibration; citizen science protocol	Mains-powered classroom units	Raised awareness; improved air exchange behaviors through feedback loops	Sensor drift; variation in user-led setup and placement
[15]	Custom LoRa-enabled prototype (RnProbe)	Simulated lab limited field setting	LoRaWAN	Laboratory validation against Alpha Guard	Solar-assisted battery	Demonstrated edge-processing and anomaly detection; low-power architecture	Prototype status; no extensive field validation

**Table 2:** Summary of recent IoT-Integrated radon monitoring studies (2020–2025).

Study	Sensor Type/Modality	Deployment Context	Connectivity/Link	Validation Method	Power/Autonomy	Key Findings	Limitations
[25]	Soil radon detectors with minute-level logging	Outdoor, seismic fault zone	Cellular IoT, cloud server	Seismic correlation and anomaly detection	Remove, solar-backed	~84% prediction sensitivity, 2.65-day lead time	Non-health applications, indoor calibration not included
[26]	Integrated indoor radon sensors in smart buildings	Residential building	Cloud-linked with web access	Internal calibration routines	Mains powered, autonomous	Fully automated monitoring system with user alerts	Deployment in one building; generalizability untested
[27]	Radon sensors in groundwater stations	Seismic/geological zones	NB-IoT	Time-series validation	Low-power, battery-based	Remote radon monitoring feasible in harsh terrain	Indoor use not studied; long-term drift unaddressed
[28]	Underwater radon-in-water detection	Marine and groundwater monitoring	Local storage and later upload	Laboratory calibration	15+ days of autonomous operation	Demonstrated viability of remote aquatic radon monitoring	Cost and sensitivity vs. air systems not benchmarked

**Table 3:** Wireless communication protocols for IoT-Embedded radon monitoring.

Protocol	Range	Power Consumption	Bandwidth	Cost	Indoor Penetration	Suitability of Radon Monitoring	Source
LoRaWAN	Up to 15 km (rural) ~2–5 km (urban	Very low	Low (0.3–50 kbps)	Low	Moderate to good	Excellent for large-scale battery-powered, low-data deployments in remote or urban schools	[55,56,57,58]
NB-IoT	Up to 10 km	Low to Moderate	Moderate (up to 250 kbps)	Medium	Excellent	Suitable for urban radon networks with good penetration through walls and basements	[59,60,61,62]
Wi-Fi	<100 m (walls reduce range)	High	High (10+ Mbps)	Moderate to high	Poor to Moderate	Suitable for homes or labs with power; not practical for long-term remote monitoring.	[63,64,65,66,67,68]
BLE	<50 m	Very Low	Low to moderate (~1 Mbps)	Low	Poor	Useful for indoor point sensing; often paired with LoRa or Wi-Fi for upstream data delivery	[69,70,71,72,73]
Cellular (3G/4G/5G)	~5–20 km	High	Very high (Mbps-Gbps)	High	Excellent	Too power-intensive for distributed sensing; suitable only for gateways or mobile monitoring.	[72,74,75,76]
Sigfox	Up to 40 km (rural, ~10 km (urban)	Very Low	Very Low (~100 bps)	Low	Moderate	Power-efficient but limited bandwidth restricts real-time alerts and high-frequency sampling.	[72,74,75,76,77,78]

**Table 4:** Case studies of major IoT deployments.

Location	Nodes	Duration	Key Findings	Limitations	References
Alberta, Canada	50	6 months	Seasonal radon spikes observed during winter; HVAC cycles impacted radon levels	Short-duration limited longitudinal analysis	[19]
Tehran, Iran	120	9 months	School basements showed concentrations above WHO action level; prompted policy changes	Inconsistent calibration across devices	[17]
Seoul, Republic of Korea	200	12 months	Identified radon hotspots in concrete-heavy buildings; material choice mattered	LoRaWAN connectivity issues in high-density areas	[13]
Boston, USA	30	3 months	Crowd-sourced sensors successfully mapped indoor air risks; public dashboards improved engagement	Calibration drift and user error in DIY setups	[18]

**Table 5:** Summary of Real-World IoT Radon Monitoring Deployments.

Setting	Project/Study	Technology	Key Takeaway
Public buildings/school	RnMonitoring (Portugal) [13]	LoRaWAN, WebGIS	Scalable, municipal-level risk mapping and dashboarding
School indoor mapping	UK School Analysis [95]	Statistical distribution modeling	Demonstrates room-to-room radon variation; supports dense sensor placement
Residential settings	RadonEye Evaluation [96]	Consumer-grade continuous monitors	Affordable long-term monitoring is viable for households
Community health programs	Northamptonshire Campaign [97]	Electret ion chamber (passive)	Mass-deployment potential in public health settings
Home vs. workplace analysis	Los Alamos Study [98]	Track-etch detectors	Home exposure dominates; justifies a holistic, cross-setting monitoring strategy.

## Data Availability

Not applicable.

## References

[1] Eggers C. (2015). Developing a National Database of Radon Test Data in Collaboration with EPA: A Pilot Project to Ascertain Feasibility. Online J. Public Health Inform..

[2] El-Araby E.H., Babeer A.M. (2013). Assessment of Indoor Radon Concentration in Different Houses in Jazan City in Saudi Arabia. Web Pub.

[3] Ferri G.M., Cavone D., Intranuovo G., Birtolo F., Tricase P., Fuso R., Vimercati L. (2018). 484 Radon and Risk of Lung Cancer in Apulia Region Southern Italy. Occup. Envrion. Med..

[4] Larsson L.S. (2014). Risk-Reduction Strategies to Expand Radon Care Planning with Vulnerable Groups. Public Health Nurs..

[5] Eising E. (2010). Correspondence (Letter to the Editor): Risk Associated with Radon Is Overestimated. Dtsch. Arztebl. Int..

[6] Shahbazi Sehrani M., Boudaqpoor S., Mirmohammadi M. (2019). Measurement of Indoor Radon Gas Concentration and Assessment of Health Risk in Tehran, Iran. Int. J. Environ. Sci. Technol..

[7] Frutos-Puerto S., Hurtado-Sanchez M., Pérez J.d.l.T., Pinilla-Gil E., Miró C. (2021). Radon Alpha Track Counting on Solid State Nuclear Track Detector by an ImageJ-Based Software Macro. Appl. Radiat. Isot..

[8] Zhao C., Zhuo W., Fan D., Yi Y., Chen B. (2014). Effects of Atmospheric Parameters on Radon Measurements Using Alpha-Track Detectors. Rev. Sci. Instrum..

[9] Sorimachi A., Nagamatsu Y., Omori Y., Ishikawa T. (2021). Comparison Experiments for Radon and Thoron Measuring Instruments at Low-Level Concentrations in One Room of a Japanese Concrete Building. Appl. Radiat. Isot..

[10] Hofmann W., Arvela H.S., Harley N.H., Marsh J.W., McLaughlin J., Röttger A., Tokonami S. (2012). 8. Variabilities and Uncertainties of Radon and Radon Progeny Exposure and Dosimetry. J. ICRU.

[11] Ruvira B., García-Fayos B., Juste B., Arnal J.M., Verdú G. (2022). Determination of the Radon Diffusion Coefficient of Thin Polyethene and Aluminium Foils Used as Single or Multilayer Configuration Barriers. Radiat. Phys. Chem..

[12] Hazar N., Karbakhsh M., Yunesian M., Nedjat S., Naddafi K. (2014). Perceived Risk of Exposure to Indoor Residential Radon and Its Relationship to Willingness to Test among Health Care Providers in Tehran. J. Environ. Health Sci. Eng..

[13] Lopes S.I., Moreira P.M., Cruz A.M., Martins P., Pereira F., Curado A. RnMonitor: A WebGIS-Based Platform for Expedite in Situ Deployment of IoT Edge Devices and Effective Radon Risk Management. Proceedings of the 2019 IEEE International Smart Cities Conference (ISC2).

[14] Marini R., Mikhaylov K., Pasolini G., Buratti C. (2022). Low-Power Wide-Area Networks: Comparison of LoRaWAN and NB-IoT Performance. IEEE Internet Things J..

[15] Pereira F., Lopes S.I., Carvalho N.B., Curado A. (2020). RnProbe: A LoRa-Enabled IoT Edge Device for Integrated Radon Risk Management. IEEE Access.

[16] Peruzzi G., Pozzebon A. (2022). Combining LoRaWAN and NB-IoT for Edge-to-Cloud Low Power Connectivity Leveraging on Fog Computing. Appl. Sci..

[17] Yousefian F., Nasiri Z., Kordi M., Marzi Y.G., Dehghani R., Mirzaei N., Janjani H., Aghaei M., Aboosaedi Z. (2024). Indoor Radon and Its Health Risk Assessment in Iran: A Comprehensive Review Study. Indoor Air.

[18] Barros N., Sobral P., Moreira R.S., Vargas J., Fonseca A., Abreu I., Guerreiro M.S. (2024). SchoolAIR: A Citizen Science IoT Framework Using Low-Cost Sensing for Indoor Air Quality Management. Sensors.

[19] Dicu T., Burghele B.D., Botoş M., Cucoș A., Dobrei G., Florică Ș., Grecu Ș., Lupulescu A., Pap I., Szacsvai K. (2021). A New Approach to Radon Temporal Correction Factor Based on Active Environmental Monitoring Devices. Sci. Rep..

[20] Emirhan M.E. (2025). Active Radon Detection Unit. Radiat. Meas..

[21] Nikesh M., Bharathi M., Naaz S.H., Srinivas T.A.S. (2025). IoT’s Biggest Headaches: Privacy, Security, and Data Management Challenges. J. IoT Secur. Smart Technol..

[22] Garcia A., Saez Y., Harris I., Huang X., Collado E. (2025). Advancements in Air Quality Monitoring: A Systematic Review of IoT-Based Air Quality Monitoring and AI Technologies. Artif. Intell. Rev..

[23] Manono Fotso Kamgang S.L., Monti M.M., Salame-Alfie A. (2023). Temporal Variation in Indoor Radon Concentrations Using Environmental Public Health Tracking Data. Health Phys..

[24] Ren H., Liu Y. (2024). A New Calibration Method for Radon Detector in Seismic Systems. Earthq. Sci..

[25] Pratama T.O., Wijatna A.B., Sunarno S., Sari H.K., Yanti R.J., Wijaya R., Waruwu M.M. (2025). Application of Continuous Radon Gas Concentration Telemonitoring for Predictive Seismic Hazard Assessment in Manado, Indonesia. Ecol. Eng. Environ. Technol..

[26] Rey J.F., Cesari M., Schoenenweid M., Montet F., Gandolla M., Bonvin L., Bourquin V., Jacot C.L., Roman J., Mahecha D. (2023). Autodigit-RAD: Towards an Automation of the Radon’s Concentration Dataflow in a New and Innovative Building. J. Phys. Conf. Ser..

[27] Jang S.H., Lee J.-K., Lee S.Y., Oh K.D. (2020). Basic Study on Development of the Radon Measurement System in Groundwater Stations for the Seismic Monitoring and Prediction. J. Korea Water Resour. Assoc..

[28] Li C., Li M., Chen G., Yu H., Zhang C., Liu W., Guo J., Zhao S., Song L., Cui X. (2023). In-Situ Detection Equipment for Radon-in-Water: Unattended Operation and Monthly Investigations. Acta Oceanol. Sin..

[29] Blanco-Novoa O., Barros P., Fraga-Lamas P., Lopes S.I., Fernández-Caramés T.M., Lopes S.I., Fraga-Lamas P., Fernándes-Camáres T.M., Dawadi B.R., Rawat D.B., Shakya S. (2023). IoT Architectures for Indoor Radon Management: A Prospective Analysis. Smart Technologies for Sustainable and Resilient Ecosystems.

[30] Visca L., Amoroso A., Calabria R., Cotto G., Crosetto A., Destefanis M.G.M., Durisi E.A., Mallamace F., Trapani P.P., Zamprotta L. (2023). Radon Detector Development Using a PIN Photodiode. J. Eur. Radon Assoc..

[31] Morishita Y., Ye Y., Mata L., Pozzi S.A., Kearfott K.J. (2020). Radon Measurements with a Compact, Organic-Scintillator-Based Alpha/Beta Spectrometer. Radiat. Meas..

[32] Anastasio A., Ambrosino F., Basta D., Bonechi L., Brianzi M., Bross A., Callier S., Cassese F., Castellini G., Ciaranfi R. (2013). The MU-RAY Experiment. An Application of SiPM Technology to the Understanding of Volcanic Phenomena. Nucl. Instrum. Methods Phys. Res. Sect. A Accel. Spectrometers Detect. Assoc. Equip..

[33] Abdullah A.N., Kamarudin K., Kamarudin L.M., Adom A.H., Mamduh S.M., Mohd Juffry Z.H., Bennetts V.H. (2022). Correction Model for Metal Oxide Sensor Drift Caused by Ambient Temperature and Humidity. Sensors.

[34] Masson N., Piedrahita R., Hannigan M. (2015). Approach for Quantification of Metal Oxide Type Semiconductor Gas Sensors Used for Ambient Air Quality Monitoring. Sens. Actuators B Chem..

[35] Dai C.-L., Liu M.-C., Chen F.-S., Wu C.-C., Chang M.-W. (2007). A Nanowire WO3 Humidity Sensor Integrated with Micro-Heater and Inverting Amplifier Circuit on Chip Manufactured Using CMOS-MEMS Technique. Sens. Actuators B Chem..

[36] Jin Z., Zhao J., Liu L., Liu F., Wang Z., Wang F., Liu J., Mou Y., Wu L., Wu X. (2024). Humidity-Independent Gas Sensors in the Detection of Hydrogen Sulfide Based on Nd_2_O_3_-Loaded In_2_O_3_ Porous Nanorods. Sens. Actuators B Chem..

[37] Kumar A., Gupta G., Bapna K., Shivagan D.D. (2023). Semiconductor-Metal-Oxide-Based Nano-Composites for Humidity Sensing Applications. Mater. Res. Bull..

[38] Chakole M., Ainchwar I., Budhe V., Babhale A., Katolkar A., Dorle S. IoT—Driven Bioelectrical Signals Detection and Monitoring System. Proceedings of the 2024 International Conference on Computational Intelligence and Computing Applications (ICCICA).

[39] Tie Y., Chen P., Ma Y. (2024). BLE Bluetooth Remote-Controlled Car Based on ESP32. Appl. Comput. Eng..

[40] Hegade V.G., Krishna Y., Menezes R. (2021). Analysis of Animals Health Condition after Post-Surgery Using LoRa Communication. Int. J. Emerg. Technol. Innov. Res..

[41] Liew Y.-H., Tan W.-H., Ooi C.-P. A Low-Power Embedded IoT System for Accurate Detection of Unauthorized Manhole Access. Proceedings of the 2024 Multimedia University Engineering Conference (MECON).

[42] Wang X., Yi X., Ding H. Battery Monitoring System Design Based on NB-IoT. Proceedings of the 2022 First International Conference on Cyber-Energy Systems and Intelligent Energy (ICCSIE).

[43] Choi H., Yun J., Park S., Kim Y., Park S. (2015). Long-Tail Watchdog Timer for High Availability on STM32F4-Based Real-Time Embedded Systems. J. Korea Multimed. Soc..

[44] Li X., Chen J. Design of Lithium Battery Management Control System Based on STM32. Proceedings of the 5th International Conference on Information Technologies and Electrical Engineering; Association for Computing Machinery.

[45] Lima E., Moraes J., Oliveira H., Cerqueira E., Zeadally S., Rosário D. (2021). Adaptive Priority-Aware LoRaWAN Resource Allocation for Internet of Things Applications. Ad Hoc Netw..

[46] Bartolín-Arnau L.M., Todoli-Ferrandis D., Sempere-Payá V., Silvestre-Blanes J., Santonja-Climent S. (2023). LoRaWAN Networks for Smart Applications in Rural Settings. IETE Tech. Rev..

[47] Kamarudin M.N.C., Ayob A., Hussain A., Ansari S., Abdolrasol M.G.M., Saad M.H.M. (2024). Review of LoRaWAN: Performance, Key Issues and Future Perspectives. J. Kejuruter..

[48] Pasandi H.B., Haqiqat A., Moradbeikie A., Keshavarz A., Rostami H., Paiva S., Lopes S.I. (2022). Low-Cost Traffic Sensing System Based on LoRaWAN for Urban Areas. Proceedings of the 1st International Workshop on Emerging Topics in Wireless.

[49] Phung K.-H., Tran H., Nguyen Q., Huong T.T., Nguyen T.-L. Analysis and Assessment of LoRaWAN. Proceedings of the 2018 2nd International Conference on Recent Advances in Signal Processing, Telecommunications & Computing (SigTelCom).

[50] Gaitán M.G., d’Orey P.M., Cecílio J., Rodrigues M., Santos P.M., Pinto L., Oliveira A., Casimiro A., Almeida L. (2022). Modeling LoRa Communications in Estuaries for IoT Environmental Monitoring Systems. IEEE Sens. J..

[51] Reyneke M., Mullins B., Reith M. (2023). LoRaWAN & The Helium Blockchain: A Study on Military IoT Deployment. Int. Conf. Cyber Warf. Secur..

[52] Marais J.M., Abu-Mahfouz A.M., Hancke G.P. (2020). A Survey on the Viability of Confirmed Traffic in a LoRaWAN. IEEE Access.

[53] Basu S.S., Sultania A.K., Famaey J., Hoebeke J. Experimental Performance Evaluation of NB-IoT. Proceedings of the 2019 International Conference on Wireless and Mobile Computing, Networking and Communications (WiMob).

[54] Ugwuanyi S., Paul G., Irvine J. (2021). Survey of IoT for Developing Countries: Performance Analysis of LoRaWAN and Cellular NB-IoT Networks. Electronics.

[55] Erbati M.M., Schiele G., Batke G. Analysis of LoRaWAN Technology in an Outdoor and an Indoor Scenario in Duisburg-Germany. Proceedings of the 2018 3rd International Conference on Computer and Communication Systems (ICCCS).

[56] Grübel J., Thrash T., Hélal D., Sumner R.W., Hölscher C., Schinazi V.R. The Feasibility of Dense Indoor LoRaWAN Towards Passively Sensing Human Presence. Proceedings of the 2021 IEEE International Conference on Pervasive Computing and Communications (PerCom).

[57] Kamanga I.A., Lyimo J.M., Kamanga I.A., Lyimo J.M. (2022). Review of LoRAWAN and the Protocol Suitability for Low Bandwidth Wireless Sensor Networks over 5G. Int. J. Sci. Res. Arch..

[58] Rahman M., Saifullah A. (2023). Boosting Reliability and Energy-Efficiency in Indoor LoRa. Proceedings of the 8th ACM/IEEE Conference on Internet of Things Design and Implementation.

[59] Andres-Maldonado P., Ameigeiras P., Prados-Garzon J., Navarro-Ortiz J., Lopez-Soler J.M. (2019). An Analytical Performance Evaluation Framework for NB-IoT. IEEE Internet Things J..

[60] Malarski K.M., Thrane J., Bech M.G., Macheta K., Christiansen H.L., Petersen M.N., Ruepp S. Investigation of Deep Indoor NB-IoT Propagation Attenuation. Proceedings of the 2019 IEEE 90th Vehicular Technology Conference (VTC2019-Fall).

[61] Sultania A., Blondia C., Famaey J. (2021). Optimizing the Energy-Latency Tradeoff in NB-IoT with PSM and eDRX. IEEE Internet Things J..

[62] Wan L., Zhang Z., Huang Y., Yan Y., Wang J. Performance Analysis of NB-IoT Technology for Indoor IoT Applications. Proceedings of the 2017 International Conference on Computer Technology, Electronics and Communication (ICCTEC).

[63] Horn B.K.P. (2022). Indoor Localization Using Uncooperative Wi-Fi Access Points. Sensors.

[64] Ramalingam S.P., Shanmugam P.K. (2022). A Comprehensive Review on Wired and Wireless Communication Technologies and Challenges in Smart Residential Buildings. Recent Adv. Comput. Sci. Commun..

[65] Sadowski S., Spachos P. (2018). RSSI-Based Indoor Localization with the Internet of Things. IEEE Access.

[66] Shashi Kiran M.R., Spoorthi Yadav M., Hemanth Kumar R.V., Thyagarajan M. Optimal Placement of Wi-Fi Access Points for Indoor Regions to Provide 2.4 GHz and 60 GHz Spectrum Using Dual Band Architecture. Proceedings of the 2018 International Conference on Advances in Computing, Communications and Informatics (ICACCI).

[67] Tung T.C.W., Burnett J. (2004). Radon Measurement Protocol for Residences with Different Ventilation Rates. Indoor Built Environ..

[68] Urazayev D., Eduard A., Ahsan M., Zorbas D. Indoor Performance Evaluation of ESP-NOW. Proceedings of the 2023 IEEE International Conference on Smart Information Systems and Technologies (SIST).

[69] Badihi B., Sheikh M.U., Ruttik K., Jäntti R. On Performance Evaluation of BLE 5 In Indoor Environment: An Experimental Study. Proceedings of the 2020 IEEE 31st Annual International Symposium on Personal, Indoor and Mobile Radio Communications.

[70] Del Carpio L.F., Di Marco P., Skillermark P., Chirikov R., Lagergren K. (2017). Comparison of 802.11ah, BLE and 802.15.4 for a Home Automation Use Case. Int. J. Wirel. Inf. Netw..

[71] Fafoutis X., Tsimbalo E., Zhao W., Chen H., Mellios E., Harwin W., Piechocki R., Craddock I. (2016). BLE or IEEE 802.15.4: Which Home IoT Communication Solution Is More Energy-Efficient?. EAI Endorsed Trans. Internet Things.

[72] Morin É., Maman M., Guizzetti R., Duda A. (2017). Comparison of the Device Lifetime in Wireless Networks for the Internet of Things. IEEE Access.

[73] Venkatesh R., Mittal V., Tammana H. Indoor Localization in BLE Using Mean and Median Filtered RSSI Values. Proceedings of the 2021 5th International Conference on Trends in Electronics and Informatics (ICOEI).

[74] Al-Sarawi S., Anbar M., Alieyan K., Alzubaidi M. Internet of Things (IoT) Communication Protocols: Review. Proceedings of the 2017 8th International Conference on Information Technology (ICIT).

[75] Krzak Ł., Macheta J., Kubaszek M., Worek C. (2023). Comparison of Wireless Data Transmission Protocols for Residential Water Meter Applications. Int. J. Electron. Telecommun..

[76] Lauridsen M., Nguyen H., Vejlgaard B., Kovacs I.Z., Mogensen P., Sorensen M. Coverage Comparison of GPRS, NB-IoT, LoRa, and SigFox in a 7800 Km^2^ Area. Proceedings of the 2017 IEEE 85th Vehicular Technology Conference (VTC Spring).

[77] Alqurashi H., Bouabdallah F., Khairullah E. (2023). SCAP SigFox: A Scalable Communication Protocol for Low-Power Wide-Area IoT Networks. Sensors.

[78] de Oliveira F.C., Rodrigues J.J.P.C., Rabêlo R.A.L., Mumtaz S. Performance Delay Comparison in Random Access Procedure for NB-IoT, LoRa, and SigFox IoT Protocols. Proceedings of the 2019 IEEE 1st Sustainable Cities Latin America Conference (SCLA).

[79] Kotrappa P., Stieff F. (2008). One Cubic Metre NIST Traceable Radon Test Chamber. Radiat. Prot. Dosim..

[80] Linzmaier D., Röttger A. (2013). Development of a Low-Level Radon Reference Atmosphere. Appl. Radiat. Isot..

[81] Xu B., Burnett W.C., Lane-Smith D., Yu Z. (2010). A Simple Laboratory-Based Radon Calibration System. J. Radioanal. Nucl. Chem..

[82] Aït-Ziane M., Allab M., Louns-Mokrani Z. (2019). Response Verification of Integrated Device Used for Radon Measurements in Air. Braz. J. Rad. Sci..

[83] Lin C.-F., Wang J.-J., Lin S.-J., Lin C.-K. (2013). Performance Comparison of Electronic Radon Monitors. Appl. Radiat. Isot..

[84] Staaf H. (2025). The Future of Green Tech: Self-Powered IoT Sensors. Open Access Gov..

[85] Rahmadani A.A., Syaifudin Y.W., Setiawan B., Panduman Y.Y.F., Funabiki N. (2025). Enhancing Campus Environment: Real-Time Air Quality Monitoring Through IoT and Web Technologies. J. Sens. Actuator Netw..

[86] Giammanco S., Bonfanti P., Neri M. (2023). Radon on Mt. Etna (Italy): A Useful Tracer of Geodynamic Processes and a Potential Health Hazard to Populations. Front. Earth Sci..

[87] Stojanovska Z., Boev B., Zunic Z.S., Ivanova K., Ristova M., Tsenova M., Ajka S., Janevik E., Taleski V., Bossew P. (2016). Variation of Indoor Radon Concentration and Ambient Dose Equivalent Rate in Different Outdoor and Indoor Environments. Radiat. Environ. Biophys..

[88] Stojanovska Z., Januseski J., Boev B., Ristova M. (2012). Indoor Exposure of Population to Radon in the FYR of Macedonia. Radiat. Prot. Dosim..

[89] Abbasi A. (2017). Modeling of Lung Cancer Risk Due to Radon Exhalation of Granite Stone in Dwelling Houses. J. Can. Res. Ther..

[90] Kitto M.E., Haines D.K., Arauzo H.D. (2009). Emanation of Radon From Household Granite. Health Phys..

[91] Barbosa S., Pereira A., Neves L. High-Frequency Variability of Radon in a Stable Indoor Environment. 5 November 2018. https://ucdigitalis.uc.pt/pombalina/item/68226.

[92] Janik M., Bossew P. (2016). Analysis of Simultaneous Time Series of Indoor, Outdoor and Soil Air Radon Concentrations, Meteorological and Seismic Data. Nukleonika.

[93] Tsapalov A., Kovler K. (2022). Temporal Uncertainty versus Coefficient of Variation for Rational Regulation of Indoor Radon. Indoor Air.

[94] Tsapalov A., Kovler K. (2018). Indoor Radon Regulation Using Tabulated Values of Temporal Radon Variation. J. Environ. Radioact..

[95] Kouroukla E., Gooding T.D. (2024). Distribution of Radon in Large Workplaces: An Analysis Performed on Radon Levels Measured in UK Schools. J. Radiol. Prot..

[96] Evans C.E., Kearfott K.J. (2025). A 291-Day Evaluation of the Performance of a Consumer-Grade Temporal Radon Detector. Health Phys..

[97] Denman A.R., Groves-Kirkby C.J., Phillips P.S., Crockett R.G.M., Woolridge A., Gillmore G.K. (2005). The Practical Use of Electrets in a Public Health Radon Remediation Campaign. J. Environ. Radioact..

[98] Whicker J., Mcnaughton M. (2009). Work to save dose: Contrasting effective dose rates from radon exposure in workplaces and residences against the backdrop of public and occupational regulatory limits. Health Phys..

[99] Excel D.S. (2024). Edge Computing: Opportunities and Challenges. World J. Adv. Res. Rev..

[100] Chatterjee A., Chaubey N., Thampi S.M., Jhanjhi N.Z. (2022). Development of Smart Sensor for IoT Based Environmental Data Analysis Through Edge Computing. Proceedings of the Computing Science, Communication and Security, Mehsana, India, 6–7 February 2023.

[101] Roostaei J., Wager Y.Z., Shi W., Dittrich T., Miller C., Gopalakrishnan K. (2023). IoT-Based Edge Computing (IoTEC) for Improved Environmental Monitoring. Sustain. Comput. Inform. Syst..

[102] Mete M.O. (2023). Geospatial Big Data Analytics For Sustainable Smart Cities. Int. Arch. Photogramm. Remote Sens. Spat. Inf. Sci..

[103] Biswal A., Subhashini J., Pasayat A.K. (2019). Air Quality Monitoring System for Indoor Environments Using IoT. AIP Conf. Proc..

[104] Hahn E.J., Haneberg W.C., Stanifer S.R., Rademacher K., Backus J., Rayens M.K. (2023). Geologic, Seasonal, and Atmospheric Predictors of Indoor Home Radon Values. Environ. Res. Health.

[105] Qiao Y. (2018). JCSE01.04 Risk Modeling for the Early Detection of Tin Miner Lung Cancer in China. J. Thorac. Oncol..

[106] Zhukovsky M., Yarmoshenko I., Onishchenko A., Malinovsky G. (2021). Prognostic Assessment of Lung Cancer Risk under Combined Action of Radon and Smoking Using an Additive-Multiplicative Risk Model. Radiatsionnaya Gygiena = Radiat. Hyg..

[107] Ptiček Siročić A., Kovač S., Stanko D., Pejak I. (2021). Dependence of Concentration of Radon on Environmental Parameters—Multiple Linear Regression Model. Environ. Eng.—Inženjerstvo Okoliša.

[108] Jacob M.T., François K., Dieu Souffit G., Modibo O.B., Yerima Abba H., Michaux K.N., Saïdou, Tokonami S. (2023). Low-Cost Radon Monitoring with Validation by a Reference Instrument. Instrum. Sci. Technol..

[109] Ladeira E.F., Jafari S.N., Pinto R.P., Silva B.M.C. RuraLTHINGS: A Novel IoT Monitoring System for Remote and Rural Regions. Proceedings of the 2024 20th International Conference on Wireless and Mobile Computing, Networking and Communications (WiMob).

[110] Hevey D., Perko T., Martell M., Bradley G., Apers S., Rovenská K.N. (2023). A Psycho-Social-Environmental Lens on Radon Air Pollutant: Authorities’, Mitigation Contractors’, and Residents’ Perceptions of Barriers and Facilitators to Domestic Radon Mitigation. Front. Public Health.

[111] Khan S.M., Gomes J., Nicol A.-M. (2022). Residents’ Perception and Worldview about Radon Control Policy in Canada: A pro-Equity Social Justice Lens. Front. Public Health.

[112] Bhat N., Ukkund S.J., Ashraf M., Acharya K., Ramegouda N.J., Puthiyillam P., Hasan M.A., Islam S., Koradoor V.B., Praveen A.D. (2023). GO/CuO Nanohybrid-Based Carbon Dioxide Gas Sensors with an Arduino Detection Unit. ACS Omega.

[113] Nath N., Kumar A., Chakroborty S., Soren S., Barik A., Pal K., de Souza F.G. (2023). Carbon Nanostructure Embedded Novel Sensor Implementation for Detection of Aromatic Volatile Organic Compounds: An Organized Review. ACS Omega.

[114] Owais T., Khater M., Al-Qahtani H. (2024). Graphene-Based MEMS Devices for Gas Sensing Applications: A Review. Micro Nanostruct..

[115] Kotrappa P., Stieff L., Stieff F. (2016). Measurement of Radon in Natural Gas and in Propane Using Electret Ion Chambers. https://www.radelec.com/publications/Kotrappa_MeasurementOfRadonInNaturalGasAndPropaneUsingEIC_2015.pdf.

[116] Nodari B., Caldara M., Re V., Fabris L. Radon Fast Detection and Environmental Monitoring with a Portable Wireless System. Proceedings of the 2015 6th International Workshop on Advances in Sensors and Interfaces (IWASI).

[117] Kaschner M., Kafka V., Marčišovský M., Staněk P., Švihra P. (2022). Monte carlo simulation of polonium ion collection in electrostatic field for the purpose of radon detector development. Radiat. Prot. Dosim..

[118] Salvi F. (2023). Mapping the Fraction of Dwellings Exceeding Radon Level Thresholds in the Lazio Region, Central Italy. Radiat. Prot. Dosim..

[119] Berquist J., Zhou L., Whyte J., Li Y., Vuotari M., Nong G. (2019). Two Case Studies on Ventilation for Indoor Radon Control.

[120] Natarajan C., Seethapathy C., Challa V.S., Balasubramaniam V. (2025). Study on the Dynamics of Radon Concentration Buildup in the Closed-Loop Measurement System with RAD7 Online Radon Monitor. Radiat. Prot. Dosim..

[121] Xia M., Ye Y.-J., Liu S.-Y. (2024). Numerical Simulations for Radon Migration and Exhalation Behavior during Measuring Radon Exhalation Rate with Closed-Loop Method. Nucl. Sci. Tech..

[122] Sharma M. (2024). Security and Compliance in Cloud ERP Systems: A Deep Dive into Workday’s Framework. Int. Sci. J. Eng. Manag..

[123] Hussein A.H.A., Jabbar K.A., Mohammed A., Jasim L. (2024). Harvesting the Future: AI and IoT in Agriculture. E3S Web Conf..

